# Caste-Dependent Interspecific Tolerance Permits Alien Reproductives to Reproduce Within Host Colonies in *Reticulitermes* Termites Under Laboratory Conditions

**DOI:** 10.3390/insects17010076

**Published:** 2026-01-09

**Authors:** Zhuang-Dong Bai, Ya-Nan Dong, David Sillam-Dussès, Rui-Wu Wang

**Affiliations:** 1 Ministry of Education Key Laboratory for Ecology of Tropical Islands, Key Laboratory of Tropical Animal and Plant Ecology of Hainan Province, College of Life Sciences, Hainan Normal University, Haikou 571158, China; 2 School of Life Science and Technology, Northwestern Polytechnical University, Xi’an 710072, China; dyn0489@mail.nwpu.edu.cn; 3 Laboratory of Experimental and Comparative Ethology, LEEC, UR4443, University Sorbonne Paris Nord, 93430 Villetaneuse, France; drdavidsd@hotmail.com; 4 College of Life Sciences, Zhejiang University, Hangzhou 310058, China; wangrw@nwpu.edu.cn

**Keywords:** termite *Reticulitermes*, nestmate recognition, interspecific tolerance, caste-dependent aggression, interspecific infiltration

## Abstract

Nestmate recognition is the primary defense mechanism maintaining the integrity of eusocial insect colonies. While social parasitism is widespread in Hymenoptera, it is rarely documented in termites, and the behavioral boundaries preventing interspecific infiltration remain poorly understood. Here, we investigated the potential for interspecific integration between two closely related termite species under laboratory conditions. We introduced *Reticulitermes labralis* workers and reproductives (queens and kings) into orphaned groups of *R. aculabialis*. We found that host workers exhibited caste-dependent aggression: introduced workers were immediately attacked and eliminated, whereas alien reproductives were partially tolerated. Surviving alien reproductives successfully integrated into host group, receiving allogrooming and trophallactic care from host workers. Crucially, these integrated pairs produced viable eggs and larvae. Molecular analysis confirmed that the brood reared by the host workers were the genetic offspring of the introduced *R. labralis* pair, demonstrating successful “cuckoo-like” reproduction. These findings reveal that termite colony recognition is sufficiently flexible to permit the acceptance of heterospecific reproductives when native royals are absent. While field evidence remains to be discovered, our results demonstrate that the behavioral and physiological prerequisites for social parasitism exist in termites, supporting the hypothesis that close phylogenetic relatedness (Emery’s rule) facilitates the breach of social barriers.

## Simple Summary

Termite colonies are known as “closed fortresses” that fiercely attack any intruders to protect their nest. However, it is unclear whether this defense is always perfect. In this study, we tested whether termites from one species (*Reticulitermes labralis*) could successfully infiltrate and live within the colony of a different species (*Reticulitermes aculabialis*). We introduced workers, queens, and kings of *R. labralis* into orphaned groups of *R. aculabialis* in the laboratory. We found that the host termites immediately killed the intruder workers, but surprisingly tolerated the intruder queens and kings. These surviving royal intruders were fed by the host workers and successfully produced their own babies. We used molecular testing to prove that the new babies were indeed the offspring of the intruders. This study shows that termite colony defense has a “loophole”: while they recognize and kill foreign workers, they may be tricked by foreign queens and kings. This finding helps us understand how social cheating (parasitism) might evolve in insects.

## 1. Introduction

Nestmate recognition constitutes the primary immune defense of eusocial insects, allowing colonies to protect their resources and brood from competitors and predators [1,2]. In termites, this recognition system is largely mediated by cuticular hydrocarbons (CHCs) and environmental cues, typically resulting in immediate and often lethal aggression toward any non-nestmate, especially those of different species [3,4]. Consequently, widely accepted dogma suggests that termite colonies are closed societies with strict species boundaries [5]. However, the absolute rigidity of these boundaries has been challenged by observations of colony fusion in lower termites and the existence of obligate inquilines in higher termites [6,7,8]. These phenomena raise a fundamental question: Is the termite recognition system sufficiently flexible to permit the infiltration of heterospecific individuals under specific conditions?

While social parasitism, where one species exploits the social care of another, is highly diverse in the Hymenoptera (ants, bees, and wasps) [9,10,11,12], it is virtually undescribed in termites. Theoretical explanations for this disparity often cite the hemimetabolous development of termites and the lifetime presence of the royal pair, which theoretically leaves little niche space for parasitic intruders [13,14]. Nevertheless, early reviews and laboratory assays have noted instances where heterospecific termites, particularly reproductives, survived initial encounters with alien colonies [3]. Despite these intriguing historical observations, it remains unknown whether such surviving “intruders” can physiologically integrate into the host colony, exploit the host’s labor for brood care, and successfully produce viable offspring. Determining this reproductive potential is crucial for understanding whether the behavioral precursors for social parasitism exist in termites.

A potential driver for such integration is phylogenetic proximity. “Emery’s rule” posits that social parasites are often closely related to their hosts, as shared ancestry implies similar chemical profiles and communication signals, facilitating deception or tolerance [15,16]. In termites, while colony-specific odors vary, certain “royal” recognition signals (e.g., queen pheromones) appear to be evolutionarily conserved across species [17,18]. This suggests a hypothesis of caste-dependent tolerance: while host workers may aggressively reject alien workers due to divergent colony odors, they might exhibit reduced aggression toward alien reproductives that bear conserved status signals.

**Figure 1:**
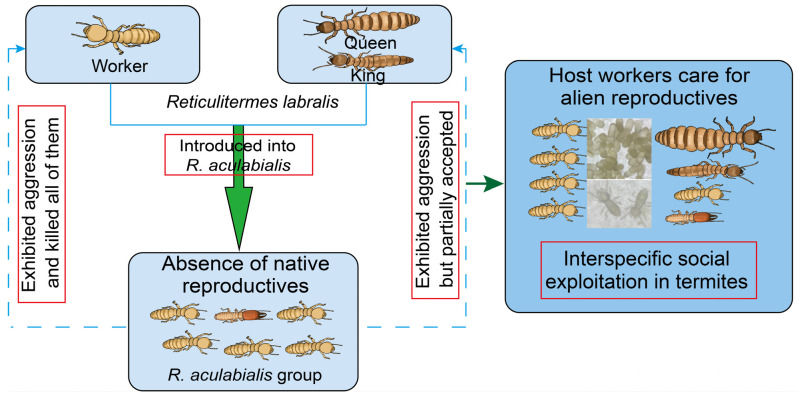
Schematic overview of interspecific infiltration observed under laboratory conditions. Diagram illustrating the caste-dependent pattern of interspecific tolerance in termites: alien workers were attacked and killed, whereas queens and kings can integrate, receive care from host workers, and reproduce successfully. The sequence of events: aggression, partial acceptance, and eventual reproductive success, reveals a full behavioral pathway consistent with an early stage of interspecific integration.

**Figure 2:**
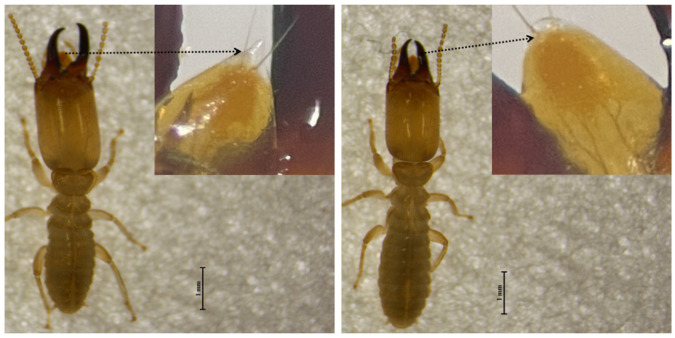
Morphological differences between the two termite species. Soldiers in *R. aculabialis* (**left**) and in *R. labralis* (**right**) showing distinct labral shapes. Insets highlight the hyaline tip of the labrum: pointed and transparent in *R. aculabialis*, oval in *R. labralis* (black dotted arrows).

To test the permeability of species boundaries and the hypothesis of caste-dependent tolerance, we examined interactions between two subterranean termites, *Reticulitermes labralis* and *R. aculabialis* (Figure 1). These species overlap in distribution in central China and share similar nesting ecology, yet they possess distinct morphological and developmental traits (Figure 2, Figures S1 and S2). We established a controlled laboratory model to simulate the intrusion of *R. labralis* individuals (workers, queens, and kings) into orphaned *R. aculabialis* groups. Unlike previous studies that primarily focused on behavioral assays or short-term survival, we monitored the long-term reproductive outcomes of these mixed associations. We utilized species-specific microsatellite markers to verify the parentage of the resulting brood. This study provides the confirmed evidence that termite reproductives can not only survive within a heterospecific colony, but also successfully exploit host workers to rear their own offspring, revealing a latent potential for parasitic-like strategies in the genus *Reticulitermes*.

## 2. Materials and Methods

### 2.1. Termite Collection and Species Identification

Field colonies of *Reticulitermes aculabialis* and *R. labralis* were collected from decaying logs at two distinct geographic locations in China: Purple Mountain, Nanjing (Jiangsu Province; 118°53′ E, 32°03′ N) and Guifeng Mountain, Xi’an (Shaanxi Province; 108°46′ E, 34°00′ N). A total of five independent colonies per species were collected to ensure genetic diversity. Species identity was confirmed using a dual approach: morphological examination of soldier castes (labrum shape) and mitochondrial cytochrome oxidase subunit II (COII) gene sequencing (Supplementary Methods and Table S1). While workers of both species are morphologically similar, they can be distinguished by significant differences in body size and head width (see Figure S1), which were used to identify individuals during behavioral observations. Stock colonies were maintained in the laboratory in plastic containers provided with moist pine wood at 25 °C and 70% relative humidity under a 14L:10D photoperiod before experimental setup.

### 2.2. Experimental Setup and Neotenic Differentiation

To establish standardized experimental units, we separated termites from stock logs. For each replicate, 200 individuals (192 workers and 8 soldiers) were housed in a 9-cm Petri dish lined with moist filter paper. This group size was chosen to mimic a functional social unit while allowing for precise observation. A total of 40 replicate groups were established for each species. Groups were maintained under the controlled conditions described above, with water and filter paper replenished weekly. Within one month of isolation, neotenic reproductives (secondary queens and kings) differentiated from nymphs or workers in the *R. labralis* groups. To standardize the reproductive status of ‘donor’ groups, we removed excess reproductives, leaving exactly one neotenic queen and one neotenic king per *R. labralis* dish. Conversely, for *R. aculabialis* groups designated as ‘recipients’ (hosts), any differentiated reproductives were strictly removed to create an orphaned, reproductive-less state.

### 2.3. Interspecific Introduction Assays

We designed an introduction assay to simulate the intrusion of alien reproductives into an orphaned colony. The experimental design consisted of three treatments: 1. Mixed Group (Experimental): One *R. labralis* worker, one queen, and one king were introduced into an orphaned *R. aculabialis* host group (*n* = 20). To minimize handling stress and physical injury, introduced individuals were gently transferred using soft entomological forceps into the center of the recipient dish. 2. Host Control (Negative Control): Orphaned *R. aculabialis* groups maintained without any introduction to monitor baseline survival and verify the absence of spontaneous differentiation (*n* = 20). 3. Conspecific Control (Positive Control): *R. labralis* colonies maintained with their own native neotenic pair to provide baseline data on reproductive output (*n* = 20).

### 2.4. Behavioral Observations and Survival Monitoring

Aggression Assays: Immediately following introduction, interactions were video-recorded using a Sony AX100E camera (Sony Corporation, Tokyo, Japan). Aggressive behavior was quantified by counting the cumulative number of biting attacks directed by host workers toward each introduced individual (worker, queen, or king) during the first 2 min of contact. Long-term Survival: Mortality of the introduced individuals was recorded at 3 h, 24 h, and daily for the first week. Once the groups stabilized (cessation of aggression), monitoring continued weekly until day 140.

Social Care Quantification: To assess social integration, we analyzed caregiving behaviors in stabilized Mixed Groups (*n* = 5) and Conspecific Controls (*n* = 5). Three 30-min video sessions were recorded for each group. We quantified the frequency of allogrooming (worker toward royal/egg) and trophallaxis (stomodeal and proctodeal; Figure S3).

### 2.5. Reproductive Assessment and Parentage Verification

The presence of eggs and larvae was monitored weekly starting from day 29 until day 140. Hatchability was calculated as the ratio of hatched larvae to the total number of eggs produced in each group. To rigorously verify that the brood produced in Mixed Groups originated from the introduced *R. labralis* parents and not from the host *R. aculabialis* workers (via parthenogenesis or undetected neotenics), we performed molecular parentage analysis. Genomic DNA was extracted from workers, eggs (*n* = 20) and larvae (*n* = 3) using a phenol-free extraction kit (Tiangen Biotech, Beijing, China). We screened six microsatellite loci (Rs 03, Rs 76, Rs 78, Ra 132, Ra 141, Ra 144; see Table S2) developed for *Reticulitermes* [19,20]. Among these, primers Ra132 and Ra141 were identified as diagnostic markers: Ra132 amplifies a ~250 bp fragment exclusively in *R. aculabialis*, while Ra 141 amplifies a ~250 bp fragment in both species (Figure S4). PCR amplification was conducted as follows: 94 °C for 5 min; followed by 30 cycles of 94 °C for 30 s, 52 °C for 30 s, and 72 °C for 30 s; and a final extension at 72 °C for 10 min. Products were visualized on 1% agarose gels to determine species origin.

### 2.6. Statistical Analysis

To account for potential non-independence among replicates originating from the same colony, we fitted linear mixed-effects models (LMMs) using the *lme4* and *lmerTest* packages in R, with colony identity included as a random effect. Differences in aggression toward introduced *R. labralis* castes (worker, queen, king) were analyzed with LMMs including caste as a fixed effect. Model syntax: lmer (attack_count ~ caste + (1|colony_ID), data = data), while mortality was analyzed using Fisher’s exact tests where data distributions did not allow for parametric modeling. Frequencies of grooming and trophallaxis were compared between host (Mixed Group) and conspecific (Control) workers using LMMs, using species identity (host vs. conspecific workers) as a fixed factor and colony ID as a random factor. Egg/larval counts and hatchability were compared between Mixed Group and Control Group using similar mixed models, while hatchability rates were also analyzed using Fisher’s exact tests where data distributions did not allow for parametric modeling. All tests were two-tailed with a significance threshold of α = 0.05. All statistical analyses and data visualization were conducted in R version 4.3.2.

## 3. Results

### 3.1. Caste-Dependent Aggression and Survival of Introduced Termites

**Figure 3:**
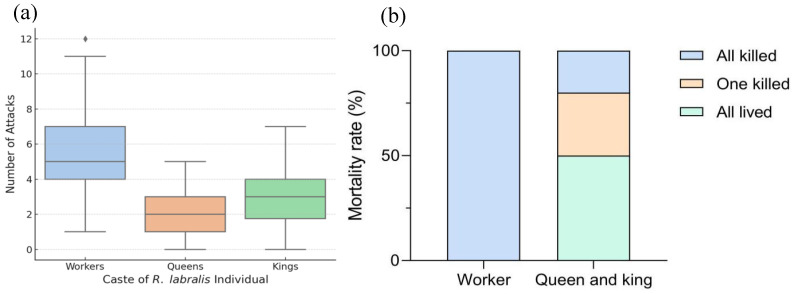
Aggression and survival of alien castes following interspecific introduction. (**a**) Number of aggressive acts (bites) by *R. aculabialis* workers toward introduced *R. labralis* workers, queens, and kings during the first 2 min. Host workers attacked alien workers significantly more frequently than reproductives (LMM, *p* < 0.001). Boxes show interquartile ranges, horizontal lines indicate medians; dots represent outliers. (**b**) Mortality outcomes of introduced individuals across 20 experimental groups. All alien workers were killed; among royal pairs, 20% of both individuals died, 30% lost one partner, and 50% survived together.

Upon introduction into orphaned *R. aculabialis* groups, host workers exhibited immediate and highly discriminatory aggression based on the caste of the *R. labralis* intruders. The frequency of biting attacks directed toward introduced workers was significantly higher than that directed toward introduced queens (LMM: *z* = 10.17, *p* < 0.001) or kings (z = 7.28, *p* < 0.001). This selective aggression resulted in distinct mortality outcomes: 100% of introduced *R. labralis* workers were killed within 24 h of introduction. In contrast, the alien reproductives exhibited significantly higher survival rates (Fisher’s exact test, *p* < 0.001; Figure 3). Specifically, in 50% of the experimental groups, both the alien queen and king survived the initial aggression and persisted for the entire 140-day observation period. In 30% of groups, one of the pair survived, while both were eliminated in only 20% of cases. These results indicate that, while species recognition cues trigger lethal aggression against foreign workers, these barriers are relaxed toward foreign reproductives. In addition, our observations found that the *R. labralis* workers died quickly after the attacks began, while it took more time for the reproductives to succumb (Table S3).

### 3.2. Behavioral Integration and Social Care Toward Alien Reproductives

**Figure 4:**
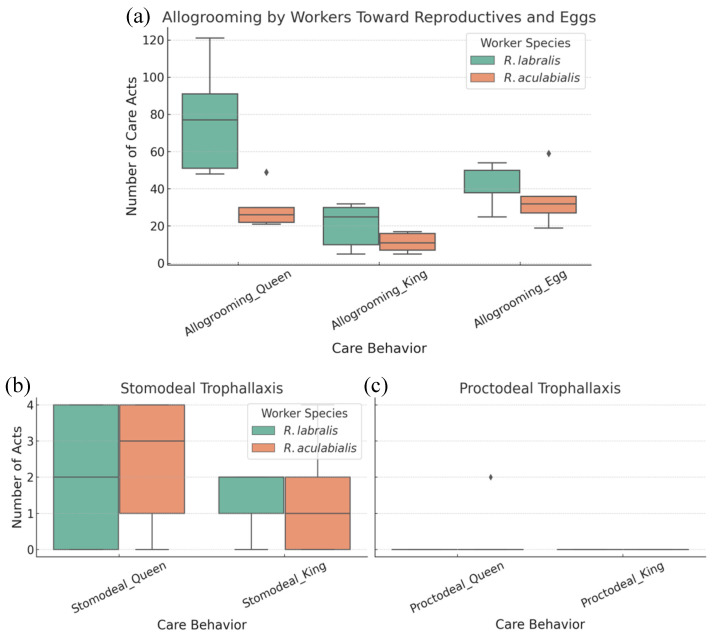
Care behavior of host and conspecific workers toward alien reproductives and eggs. Comparison of allogrooming (**a**), stomodeal trophallaxis (**b**), and proctodeal trophallaxis (**c**) performed by *R. aculabialis* (host) and *R. labralis* (conspecific) workers toward *R. labralis* queens, kings, and eggs. Host workers groomed alien reproductives and eggs significantly less often (LMM, *p* < 0.001) but showed no differences in trophallactic interactions (*p* > 0.05). Boxes show interquartile ranges; horizontal lines indicate medians, and dots indicate outliers, and dots indicate outliers.

Following the stabilization of the mixed groups (typically after one week), surviving *R. labralis* reproductives were behaviorally integrated into the *R. aculabialis* host groups. Host workers were observed performing essential caregiving behaviors, including allogrooming and trophallaxis (both stomodeal and proctodeal), toward the alien royals. The quality of care, however, varied by behavior type. The frequency of allogrooming performed by host workers toward alien queens (*z* = −4.85, *p* < 0.001) and kings (*z* = −3.87, *p* < 0.001) was significantly lower than that observed in conspecific control groups. Similarly, alien eggs received significantly less grooming from host workers compared to conspecific eggs (*z* = −3.51, *p* < 0.001). Crucially, however, nutritional integration appeared successful: the frequency of stomodeal and proctodeal trophallaxis provided by host workers to the alien queen and king did not differ significantly from that observed in conspecific *R. labralis* groups (*p* > 0.05 for all comparisons; Figure 4). This indicates that, while hygienic behavior (grooming) remains constrained by species barriers, the nutritional transfer system is permeable to support alien reproductives. Furthermore, host workers were observed actively relocating alien eggs and larvae to safety piles upon disturbance, indicating behavioral acceptance of the brood (e.g., by opening the lid of the Petri dish; Videos S1 and S2).

### 3.3. Reproductive Success and Parentage Verification of the Brood

**Figure 5:**
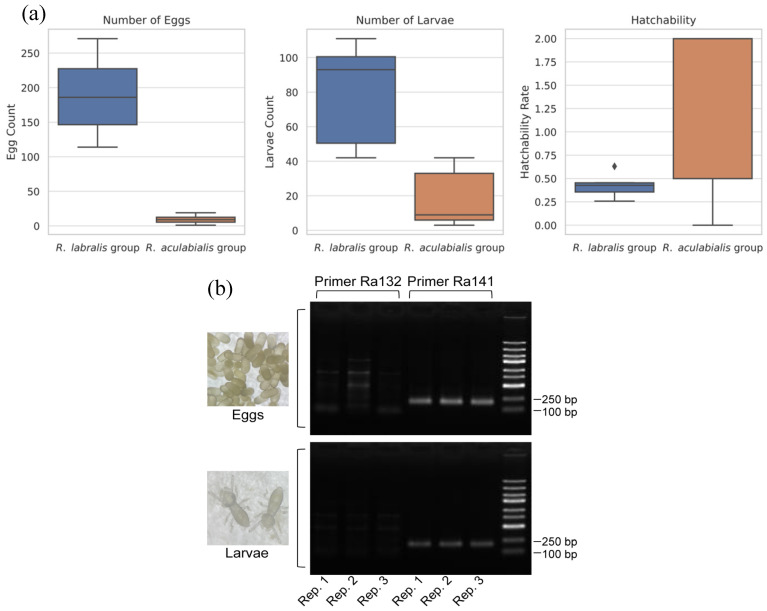
Reproductive output and parentage of offspring produced in mixed group. (**a**) Numbers of eggs (**left**), larvae (**middle**), and hatchability (**right**) in *R. aculabialis* host groups containing *R. labralis* reproductives compared with pure *R. labralis* control groups. Egg and larval numbers were lower in mixed groups (*p* < 0.001), whereas hatchability was slightly higher (*p* = 0.017). Boxes show interquartile ranges; horizontal lines indicate medians, and dots indicate outliers, and dots indicate outliers. (**b**) PCR amplification results for eggs and larvae from mixed group using diagnostic primers Ra132 (produced bands only in *R. aculabialis*) and Ra141 (amplified in both species). Three independent replicates (Rep. 1–3) show that the fragments of approximately 250 bp were successfully amplified using primer Ra141, but not with Ra132, confirming that offspring originated from the introduced *R. labralis* reproductives.

Physiological integration culminated in successful reproduction. In mixed groups where the royal pair survived, egg laying commenced on day 29, with larvae first observed on day 114. By day 140, brood was present in experimental groups, confirming that alien reproductives could exploit host resources to produce offspring. Quantitatively, the reproductive output in mixed groups was lower than in optimized conspecific controls. The total number of eggs (*z* = −23.32, *p* < 0.001) and larvae (*z* = −5.45, *p* < 0.001) was significantly reduced compared to pure *R. labralis* colonies. However, the hatchability of eggs in mixed groups was slightly higher than in controls (z = 2.38, *p* = 0.017; Figure 5a), suggesting that the surviving brood received effective developmental care from host workers.

To rule out the possibility of parthenogenesis by host workers or the presence of undetected host neotenics, we determined the genetic origin of the brood using species-specific microsatellite markers. The diagnostic primer Ra 132 amplifies a ~250 bp fragment in *R. aculabialis* but yields no product in *R. labralis*, while primer Ra141 amplifies a fragment in both species (Figure S5). PCR analysis of eggs and larvae collected from the mixed experimental groups showed consistent amplification with the universal primer Ra 141 but, crucially, no amplification with the host-specific primer Ra 132 (Figure 5b). This molecular profile matches that of the introduced *R. labralis* parents and differs from the *R. aculabialis* host profile. These results definitively confirm that the brood reared by *R. aculabialis* workers were the biological offspring of the introduced *R. labralis* reproductives.

## 4. Discussion

Our results demonstrate that the species boundaries in *Reticulitermes* are not absolute, but are instead conditionally permeable. The striking finding is the sharp contrast in host aggression: while *R. labralis* workers were immediately recognized and eliminated by *R. aculabialis* hosts, alien queens and kings were frequently tolerated and integrated. This pattern suggests that colony recognition cues are caste-specific [21,22]. In termites, worker recognition relies heavily on cuticular hydrocarbons (CHCs), which are species- and colony-specific [1]. The lethal aggression toward alien workers confirms that *R. aculabialis* maintains a functional “reject-foreign” system. However, the acceptance of alien reproductives implies that they may lack these specific rejection cues or, more likely, possess “royal recognition signals” that override species identity. Queen pheromones are known to be evolutionarily conserved across termite species [17]. We hypothesize that *R. labralis* reproductives exploit this sensory bias, a “Royal Trojan Horse” mechanism [23], where conserved royal signals suppress host aggression, allowing them to bypass the guard of *R. aculabialis* workers. This phenomenon aligns with “Emery’s rule”, where close phylogenetic relatedness facilitates social integration through shared chemical communication channels [16].

Furthermore, the survival of alien reproductives in our experiments may not be attributed solely to the host workers’ recognition of conserved royal pheromones; it likely also depends on the reproductives’ behavioral passivity. Similar “peaceful infiltration” strategies are characteristic of inquiline ants, whose queens exhibit submissive postures and reduced mobility to minimize host aggression [24]. In termites, such behavioral quiescence of reproductives may suppress the host’s recognition, triggering cues or delaying aggressive responses. This aligns with the hypothesis that early-stage parasites evolve appeasement behaviors rather than overt mimicry, behaviors that allow temporary coexistence and reduce the cost of direct conflict [25,26]. Our observations further revealed that host workers provided trophallaxis and allogrooming to alien reproductives, albeit at reduced levels compared to conspecifics. These behaviors are key components of social immunity and recognition maintenance [27,28,29]. Their occurrence toward heterospecific reproductives suggests a degree of behavioral generalization, an inherent flexibility in the social circuitry of termite workers that may inadvertently facilitate parasitism.

A key criticism of laboratory introduction assays is their lack of ecological realism, particularly given that interspecific social parasitism has not yet been documented in termites in the wild. However, our findings offer a plausible mechanism for this apparent absence. The transient nature of the association observed in our study indicates that such interactions may be ephemeral. During swarming events, alates may occasionally encounter foreign colonies that experience royal turnover, although the frequency is low. Under such conditions, the use of conserved royal recognition signals, together with behavioral passivity, may facilitate their integration into the colonies. Because host workers are short-lived, such associations could, in principle, lead to a gradual demographic shift toward offspring produced by the invading reproductives. This process may help explain why mixed-species phases are difficult to detect in natural populations. Moreover, the value of this study lies not in documenting a common field phenomenon but in revealing the physiological and behavioral capacities of the termite social system. Our results show that biological barriers (such as recognition-mediated aggression) are not the primary factors preventing parasitism between these species. When these ecological barriers are breached, as simulated in our experiment, the social barriers (recognition) are surprisingly fragile [30]. This distinction between ecological constraints and physiological capacity is vital for understanding how social parasites might evolve if environmental shifts force species into closer contact [31].

The incipient parasitism observed in *Reticulitermes*, where foreign reproductives exploit a stressed or orphaned host colony, suggests a potential parallel with the hypothetical early stages of inquilinism evolution (social parasitism without worker caste) [32]. In ants, temporary social parasitism is often a stepping stone to obligate inquilinism [33,34,35]. Our findings suggest a similar evolutionary potential exists in termites. If a mutation or environmental change aligned the swarming schedules of these species, *R. labralis* queens landing near orphaned *R. aculabialis* foraging groups could theoretically infiltrate and parasitize them [36].

## 5. Conclusions

In conclusion, our results show that the biological barriers between two sympatric *Reticulitermes* species are conditionally permeable. We demonstrate a distinct pattern of caste-dependent interspecific tolerance: while the recognition system strictly eliminates alien workers, it fails to reject alien reproductives. This vulnerability is likely driven by a “Royal Trojan Horse” mechanism, where conserved royal pheromones and behavioral passivity allow intruders to bypass host aggression. Crucially, the successful production of hybrid-reared offspring confirms that the physiological capacity for social parasitism–like exploitation exists in termites, even if it is currently suppressed by ecological constraints in nature. These findings challenge the traditional view of termite colonies as closed societies and suggest that the evolutionary precursors for social parasitism, specifically, the exploitation of host labor by close relatives (Emery’s rule), are latent within the termite lineage.

## Data Availability

The original contributions presented in this study are included in the article/Supplementary Materials. Further inquiries can be directed to the corresponding author.

## Supplementary Materials

The following supporting information can be downloaded at: https://www.mdpi.com/article/10.3390/insects17010076/s1. Figure S1: Morphological differences between soldiers of *Reticulitermes aculabialis* and *R. labralis*; Figure S2: Field microhabitat distribution and interspecific interaction between *R. aculabialis* and *R. labralis*; Figure S3: Care behaviors exhibited by host *R. aculabialis* workers toward alien *R. labralis* reproductives; Figure S4: Species-specific PCR profiles of termite workers; Figure S5: PCR verification of offspring parentage; Table S1: BLAST results of mitochondrial COII sequences of collected samples against NCBI GenBank database; Table S2: Pairs of microsatellite loci primers used; Table S3: Survival trajectories of introduced *R. labralis* queens (first symbol) and kings; Supplemental Data S1: Results from 20 additional interspecific groups established under identical experimental conditions; Supplementary Methods: Species identification and COII sequencing; Video S1: Host workers transporting alien eggs following disturbance; Video S2: Host workers relocating alien larvae following disturbance. References [19,20,37,38,39] are cited in the Supplementary Materials.
