# Supplementary material for: Caste-Dependent Interspecific Tolerance Permits Alien Reproductives to Reproduce Within Host Colonies in Reticulitermes Termites Under Laboratory Conditions
Source: Insects. 2026 Jan 9;17(1):76. doi: 10.3390/insects17010076 (PMC12842407; doi:10.3390/insects17010076)
Supplement: Supplementary file 1 [file insects-17-00076-s001.zip › Supplementary materials and methods.v8.pdf]

Supplementary materials and methods

Supplementary materials

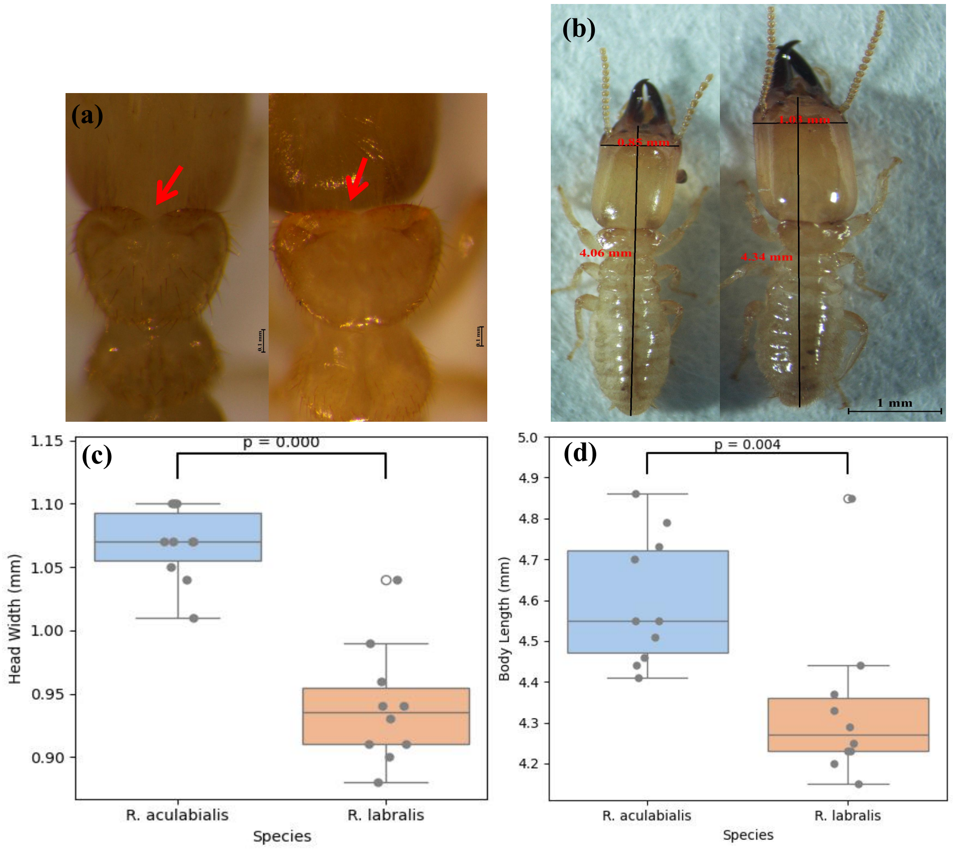

Figure S1: Morphological differences between soldiers of *Reticulitermes aculabialis* and *R. labralis*. (a) Dorsal view showing the pronotum shape: the *R. labralis* soldier (right) displays a deeper curvature than *R. aculabialis* soldiers (left). Red arrows indicate the curvature of the lateral margins of the pronotum. (b) Lateral view illustrating differences in head width and body length of *R. aculabialis* soldiers. (c, d) Boxplots showing that both head width and body length are significantly greater in *R. aculabialis* soldiers than in *R. labralis* soldiers ($p < 0.001$).

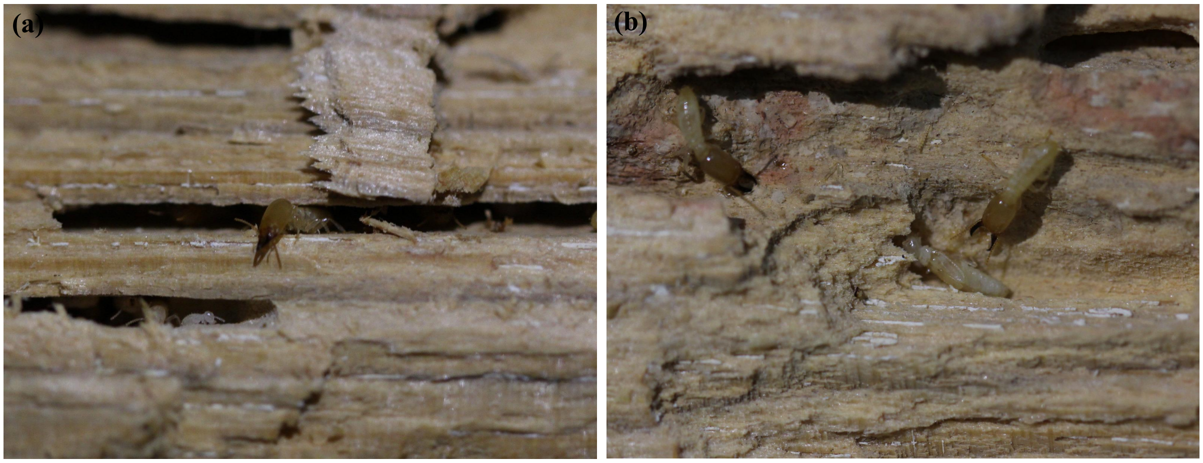

Figure S2: Field microhabitat distribution and interspecific interaction between *R. aculabialis* and
R. labralis. (a) the two species occupied different layers within the same piece of decayed wood, revealing overlapping nesting sites with partial spatial segregation. (b) Upon encountering a *R. labralis* nymph, a soldier of *R. aculabialis* initiated aggression, indicating species-specific recognition and defensive behavior.

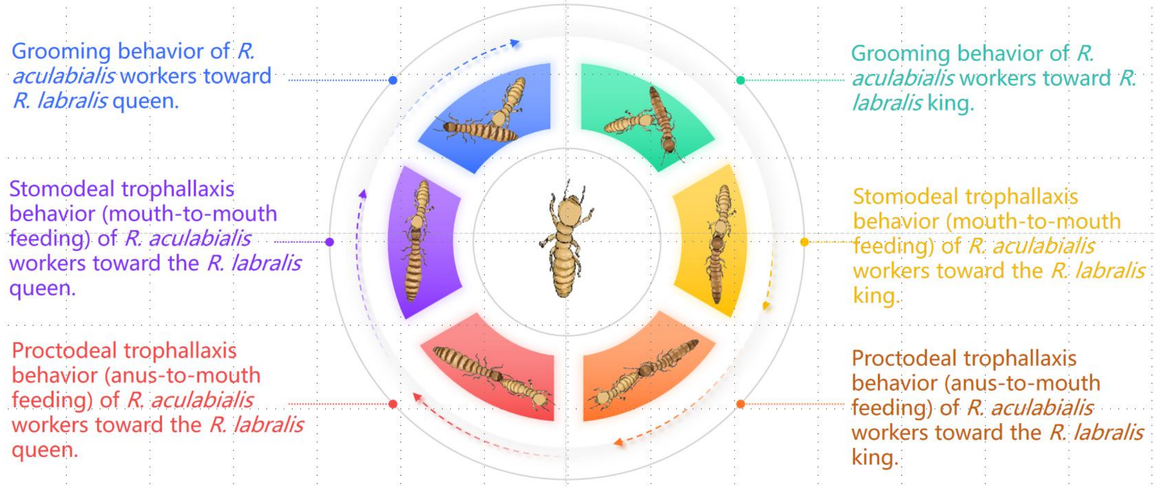

Figure S3: Care behaviors exhibited by host *R. aculabialis* workers toward alien *R. labralis* reproductives. Diagram summarizing six caregiving behaviors directed at the introduced king and queen: grooming, stomodeal trophallaxis (mouth-to-mouth feeding), and proctodeal trophallaxis (anus-to-mouth feeding). Behaviors toward the queen (blue-red) are shown on the left half; those toward the king (teal-orange) on the right. These interactions reflect the behavioral acceptance and integration of alien reproductives within host group.

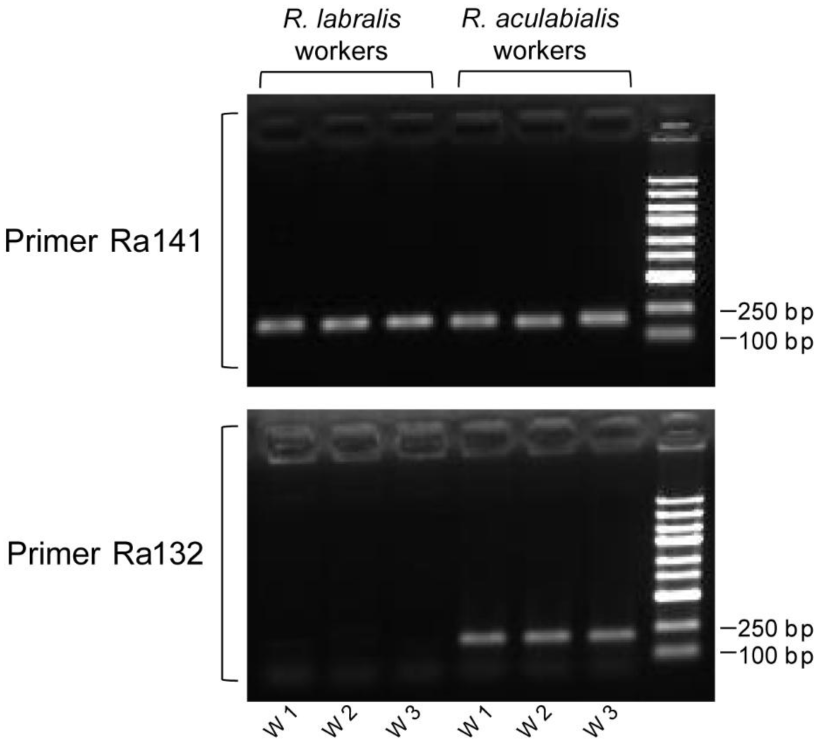

Figure S4: Species-specific PCR profiles of termite workers. PCR amplification of three workers per species using primers Ra 132 and Ra 141. A 250 bp band appeared in both species with Ra 141, whereas Ra 132 yielded a band only in *R. aculabialis*, confirming primer specificity.

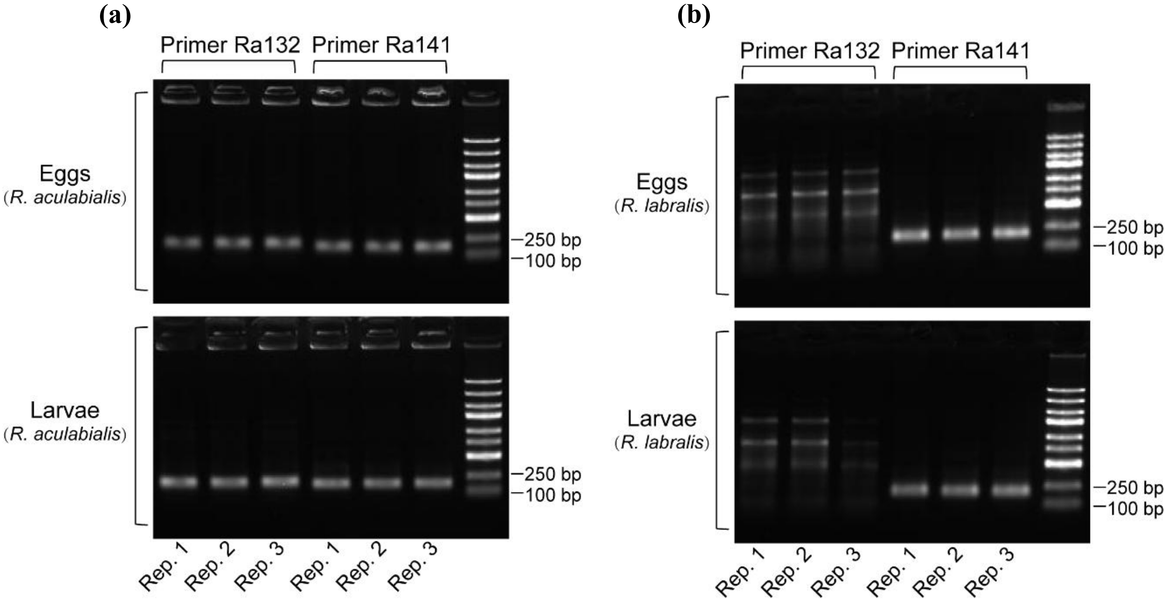

Figure S5: PCR verification of offspring parentage. (a) *R. aculabialis* samples and (b) *R. labralis* samples. The primer Ra 141A amplified a ~250 bp band in both species; Ra 132 produced a band only in *R. aculabialis.* Each primer was tested in triplicate.

Table S1. BLAST results of mitochondrial COII sequences of collected samples against NCBI GenBank database.

Sample ID	Putative Species	Best Match Description	GenBank Accession	Query Coverage (%)	Identity (%)
Worker_1	*Reticulitermes aculabialis*	*R. aculabialis* isolate HZ1 COII gene	JX142171.1	93%	99.46%
Worker_2	*Reticulitermes aculabialis*	*R. aculabialis* isolate HZ1 COII gene	JX142171.1	91%	99.46%
Worker_3	*Reticulitermes aculabialis*	*R. aculabialis* isolate HZ1 COII gene	JX142171.1	91%	99.46%
Worker_4	*Reticulitermes aculabialis*	*R. aculabialis* isolate HZ1 COII gene	JX142171.1	97%	99.31%
Worker_5	*Reticulitermes aculabialis*	*R. aculabialis* isolate HZ1 COII gene	JX142171.1	96%	99.04%
Worker_6	*Reticulitermes labralis*	*R. labralis* isolate AH1 COII gene	KU746842.1	93%	97.07%
Worker_7	*Reticulitermes labralis*	*R. labralis* isolate AH1 COII gene	KU746842.1	95%	96.60%
Worker_8	*Reticulitermes labralis*	*R. labralis* isolate AH1 COII gene	KU746842.1	92%	100.00%
Worker_9	*Reticulitermes labralis*	*R. labralis* isolate AH1 COII gene	KU746842.1	98%	96.61%
Worker_10	*Reticulitermes labralis*	*R. labralis* isolate AH1 COII gene	KU746842.1	92%	96.39%

The Table showing 91%-98% query coverage and 96.4%-100% identity to published *Reticulitermes* sequences (accessions JX142171.1, KU746842.1), corroborating morphological identification.

Primer	Primer sequences (5'-3')	Core repeat unit	Size
Rs 03	TCCTGACTGTACAAAGAAAAGTGG<br>TGGCATCAAGCTACGTATTCA	$(CT)_9$	233
Rs 76	AATCCGGGGAATTTCTTGAC<br>CTGCATAACGATGTCTGCGT	$(AGTT)_8$	175-180
Rs 78	GCTTCTCAAGAAGGACTGTGC<br>GCCCCAGTTGAGATATGGAA	$(AGTT)_7$	169-178
Ra 132	GATTGGTTTCCTCCGAATCA<br>AAAGACTACTGCCACCGGG	$(TTA)_{14}$	201-213
Ra 141	CACATTTGAGGTTCGCAAGA<br>GCCAGAAGGCCAATTACAGA	$(TTA)_8$	165-210
Ra 144	CAAATAGAGCTCCGTGTTTCG<br>CCATAGAAACCTCCGAAAGG	$(TTAG)_7$	148-184

Table S2 Pairs of microsatellite loci primers used following Dang et al., (2017), Dronnet, Bagnères, Juba, & Vargo, (2004), and Vargo & Henderson, (2000).

	3 hours	Day 1	Day 3	Day 5	Day 7	Day 15	Day 29	Day 63	Day 114	Day 137	Day 140
Group 1	(+, +)	(+, +)	(+, +)	(+, +)	(+, +)	(+, +)	(+, +)	(+, +)	(+, +)	(+, +)	(+, +)
Group 2	(+, +)	(+, +)	(+, +)	(+, +)	(+, +)	(+, +)	(+, +)	(+, +)	(+, +)	(+, +)	(+, +)
Group 3	(+, +)	(+, +)	(+, +)	(+, +)	(+, +)	(+, +)	(+, +)	(+, +)	(+, +)	(+, +)	(+, +)
Group 4	(+, +)	(+, −)	(+, −)	(+, −)	(−, −)	(−, −)	(−, −)	(−, −)	(−, −)	(−, −)	(−, −)
Group 5	(+, +)	(+, +)	(+, +)	(+, +)	(+, +)	(+, +)	(+, +)	(+, +)	(+, +)	(+, +)	(+, +)
Group 6	(+, +)	(+, +)	(+, +)	(+, +)	(+, +)	(+, +)	(+, +)	(+, +)	(+, +)	(+, +)	(+, +)
Group 7	(+, +)	(+, +)	(+, +)	(+, +)	(+, +)	(+, +)	(+, +)	(+, +)	(+, +)	(+, +)	(+, +)
Group 8	(+, +)	(+, +)	(+, +)	(+, +)	(+, +)	(+, +)	(+, +)	(+, +)	(+, +)	(+, +)	(+, +)
Group 9	(+, +)	(+, +)	(+, +)	(+, +)	(+, +)	(+, +)	(+, +)	(+, +)	(+, +)	(+, +)	(+, +)
Group 10	(+, +)	(+, +)	(+, +)	(+, +)	(+, +)	(+, +)	(+, +)	(+, +)	(+, +)	(+, +)	(+, +)
Group 11	(+, +)	(−, +)	(−, +)	(−, +)	(−, +)	(−, +)	(−, +)	(−, +)	(−, +)	(−, +)	(−, +)
Group 12	(+, −)	(+, −)	(+, −)	(+, −)	(+, −)	(+, −)	(+, −)	(+, −)	(+, −)	(+, −)	(+, −)
Group 13	(+, −)	(+, −)	(+, −)	(+, −)	(+, −)	(+, −)	(+, −)	(+, −)	(+, −)	(+, −)	(+, −)
Group 14	(−, +)	(−, −)	(−, −)	(−, −)	(−, −)	(−, −)	(−, −)	(−, −)	(−, −)	(−, −)	(−, −)
Group 15	(+, +)	(+, +)	(+, +)	(+, +)	(+, +)	(+, +)	(+, +)	(+, +)	(+, +)	(+, +)	(+, +)
Group 16	(+, +)	(−, +)	(−, +)	(−, +)	(−, +)	(−, +)	(−, +)	(−, +)	(−, +)	(−, +)	(−, +)
Group 17	(−, +)	(−, −)	(−, −)	(−, −)	(−, −)	(−, −)	(−, −)	(−, −)	(−, −)	(−, −)	(−, −)
Group 18	(+, +)	(−, +)	(−, +)	(−, +)	(−, +)	(−, +)	(−, +)	(−, +)	(−, +)	(−, +)	(−, +)
Group 19	(+, +)	(+, +)	(+, +)	(+, +)	(+, +)	(+, +)	(+, +)	(+, +)	(+, +)	(+, +)	(+, −)
Group 20	(+, +)	(−, −)	(−, −)	(−, −)	(−, −)	(−, −)	(−, −)	(−, −)	(−, −)	(−, −)	(−, −)

Table S3 Survival trajectories of introduced *R. labralis* queens (first symbol) and kings (second symbol; '+' = alive, '-' = dead) with *R. aculabialis* host groups during 140 days.

Supplemental data S1

To verify the reproducibility of behavioral outcomes, 20 additional interspecific groups were established under identical conditions. In each group, one *R. labralis* worker, king, and queen were introduced into an orphaned *R. aculabialis* group containing only workers and soldiers. Consistent with the main experiment, all alien workers were killed, but all introduced reproductives survived. Eggs were observed in 16 of 20 groups. The table below presents the number of eggs, larvae, and workers recorded weekly over a 101-day period.

	Day 53	Day 60	Day 67	Day 74	Day 81	Day 91	Day 101
	Number of eggs, larvae						Number of eggs, larvae, and workers
Group 1	0 / 0	0 / 0	0 / 0	0 / 0	0 / 0	0 / 0	0 / 0 / 0
Group 2	6 / 0	0 / 0	0 / 0	7 / 0	6 / 0	14 / 0	0 / 0 / 0
Group 3	11 / 0	14 / 0	9 / 1	11 / 3	0 / 1	0 / 0	0 / 0 / 0
Group 4	41 / 0	49 / 1	43 / 0	17 / 0	10 / 3	0 / 1	11 / 1 / 0
Group 5	74 / 0	73 / 1	55 / 16	22 / 7	17 / 5	26 / 0	52 / 0 / 0
Group 6	33 / 3	30 / 4	20 / 1	6 / 1	0 / 0	4 / 0	0 / 0 / 0
Group 7	0 / 0	0 / 0	0 / 0	0 / 0	0 / 0	0 / 0	15 / 0 / 0
Group 8	45 / 4	30 / 8	28 / 6	1 / 7	0 / 5	14 / 5	35 / 5 / 1
Group 9	36 / 18	34 / 17	23 / 20	0 / 24	0 / 21	12 / 21	0 / 22 / 6
Group 10	6 / 1	0 / 0	0 / 0	0 / 0	0 / 0	0 / 0	0 / 0 / 0
Group 11	0 / 0	0 / 0	0 / 0	0 / 0	0 / 0	0 / 0	0 / 0 / 0
Group 12	19 / 0	7 / 2	10 / 2	2 / 0	0 / 1	0 / 1	0 / 0 / 0
Group 13	70 / 1	55 / 2	47 / 8	14 / 0	6 / 1	16 / 0	14 / 0 / 0
Group 14	6 / 7	0 / 9	0 / 5	0 / 3	0 / 1	13 / 1	28 / 1 / 0
Group 15	0 / 0	0 / 0	0 / 0	0 / 0	0 / 0	0 / 0	0 / 0 / 0
Group 16	0 / 0	6 / 3	0 / 3	1 / 1	1 / 2	0 / 3	0 / 2 / 0
Group 17	17 / 3	15 / 12	16 / 15	1 / 12	10 / 13	15 / 13	17 / 15 / 3
Group 18	39 / 4	15 / 6	0 / 5	0 / 0	8 / 0	20 / 0	23 / 0 / 0
Group 19	12 / 0	4 / 0	2 / 0	0 / 2	0 / 1	0 / 1	0 / 1 / 0
Group 20	0 / 2	6 / 3	2 / 1	0 / 2	0 / 2	0 / 0	3 / 0 / 0

Supplementary methods

Species identification and COII sequencing

In addition to morphological diagnosis, species identity was verified by mitochondrial COII gene analysis. Genomic DNA was extracted using a Tiangen Biotech kit (Tiangen Biotech [Beijing] Co., LTD.). PCR amplification employed primers COII-F (5'-CAGATAAGTGCATTGGATTT-3') and COII-R (5'-GTTTAAGAGACCATTACTTA-3') (Miura, Roisin, & Matsumoto, 2000; Simon et al., 1994). Each 50 µl reaction contained 0.5 µM of each primer (Zhongke Yutong Biological Technology Co., LTD.), 25 µl of Taq mix (Biological Engineering Shanghai Co., LTD.), and 50 ng of template DNA. Thermal cycling conditions were as follows: (a) pre-denaturation at 94°C for 5 minutes; (b) 30 cycles of denaturation at 94°C for 30 seconds, annealing at 52°C for 30 seconds, and extension at 72°C for 30 seconds; (c) a final extension at 72°C for 5 minutes. PCR products were electrophoresed on a 1% agarose gels and visualized with a Tanon Gel Imaging System (Tanon Science & Technology, Shanghai, China). Sequencing was performed by Tsingke Biotechnology (Beijing, China), and the resulting sequences were aligned and compared with reference data using NCBI BLAST.

References

Dang, Y.-L., Zhang, H.-G., Meng, Y.-F., Zhang, M., Zhao, S., You, P., . . . Xing, L.-X. (2017). Isolation and Characterization of Polymorphic Microsatellite Markers for Two Subterranean Termites. *Sociobiology, 64*(3), 352-355.

Dronnet, S., Bagnères, A. G., Juba, T. R., & Vargo, E. L. (2004). Polymorphic microsatellite loci in the European subterranean termite, *Reticulitermes santonensis* Feytaud. *Molecular Ecology Notes, 4*(1), 127-129.

Miura, T., Roisin, Y., & Matsumoto, T. (2000). Molecular phylogeny and biogeography of the nasute termite genus *Nasutitermes* (Isoptera: Termitidae) in the Pacific tropics. *Molecular Phylogenetics and Evolution, 17*(1), 1-10.

Simon, C., Frati, F., Beckenbach, A., Crespi, B., Liu, H., & Flook, P. (1994). Evolution, weighting, and phylogenetic utility of mitochondrial gene sequences and a compilation of conserved polymerase chain reaction primers. *Annals of the Entomological Society of America, 87*(6), 651-701.

Vargo, E., & Henderson, G. (2000). Identification of polymorphic microsatellite loci in the Formosan subterranean termite *Coptotermes formosanus* Shiraki. *Molecular ecology, 9*(11), 1935-1938.
